# A critical evaluation of Nigeria’s response to the first wave of COVID-19

**DOI:** 10.1186/s42269-022-00729-9

**Published:** 2022-02-24

**Authors:** Ezekiel Damilare Jacobs, Malachy Ifeanyi Okeke

**Affiliations:** 1 Department of Microbiology, University of Ibadan, Ibadan, Oyo State Nigeria; 2 Department of Natural and Environmental Sciences, Biomedical Science Concentration, American University of Nigeria, 98 Lamido Zubairu Way, PMB 2250, Yola, Adamawa State Nigeria

**Keywords:** COVID-19, Nigeria, COVID-19 response, COVID-19 first wave, Reproduction number, Non-pharmaceutical intervention, Clinical intervention, Nigeria Centre for Disease Control

## Abstract

**Background:**

The first wave of the Coronavirus Disease 2019 (COVID-19) pandemic began when the first index case was reported in Nigeria on the 27th of February 2020, and since then, more than 68,000 cases of the disease were confirmed, with 1173 deaths as of November 30, 2020.

**Main body:**

Daily situation reports from the Nigeria Centre for Disease Control spanning February 27–November 30, 2020, were fully considered in this review. Further literature search was performed using PubMed and Google Scholar databases for articles related to response measures adopted by Nigeria. The instantaneous reproduction number (*R*) was then estimated as a metric to investigate the non-pharmaceutical intervention measures. Nigeria responded to COVID-19 pandemic by implementing anti-COVID-19 mitigation strategies in travel restrictions, social distancing, source control, contact tracing, self-isolation, and quarantine, as well as in clinical interventions. Our epidemiological model estimated the *R*-value of more than 1.0 in Nigeria and in each of all the 36 states and the Federal Capital Territory.

**Conclusion:**

Nigeria implemented containment and mitigation measures in response to the first wave of COVID-19 and these measures may have contributed to the mild COVID-19 outcome in Nigeria compared to the global trend. However, inadequate PCR testing capacity, lack or suboptimal utilization of epidemic metrics like the virus reproduction number (*R*) to inform decision making, and premature easing of lockdown measures among others were major challenges to the effective implementation of the COVID-19 response measures.

## Background

Severe Acute Respiratory Syndrome CoronaVirus-2 (SARS-CoV-2), which belongs to the *betacoronavirus* subgroup B is the etiological agent of the highly contagious Coronavirus Disease 2019 (COVID-19) which has ravaged the world (Lupia et al. 2020; Di Nardo et al. 2020). SARS-CoV-2 is a single-stranded, positive-sense RNA virus, with size ranging from 80 to 120 nm and possesses a genomic proofreading mechanism which prevents the virus from accumulating mutations that could weaken it (Di Nardo et al. 2020; Wang et al. 2020a; Cyranoski 2020). The spike-like glycoprotein protuberance on its envelope facilitates the binding and penetration of the protective membrane of host cells (Di Nardo et al. 2020; Wang et al. 2020a). This virus is easily spread among humans either through tiny droplet infection when people sneeze, cough, or talk, or by contacts with contaminated surfaces; and in the process causing mild to severe respiratory symptoms (Di Nardo et al. 2020). However, the disease may also present with a highly diverse clinical spectrum (Di Nardo et al. 2020; Wang et al. 2020a). Although there were no vaccines at the early stages of COVID-19 all over the world, several management therapies were in place, which was aimed at ameliorating the symptoms and also, complement the body’s natural immunity (Wang et al. 2020a).

**Fig. 1:**
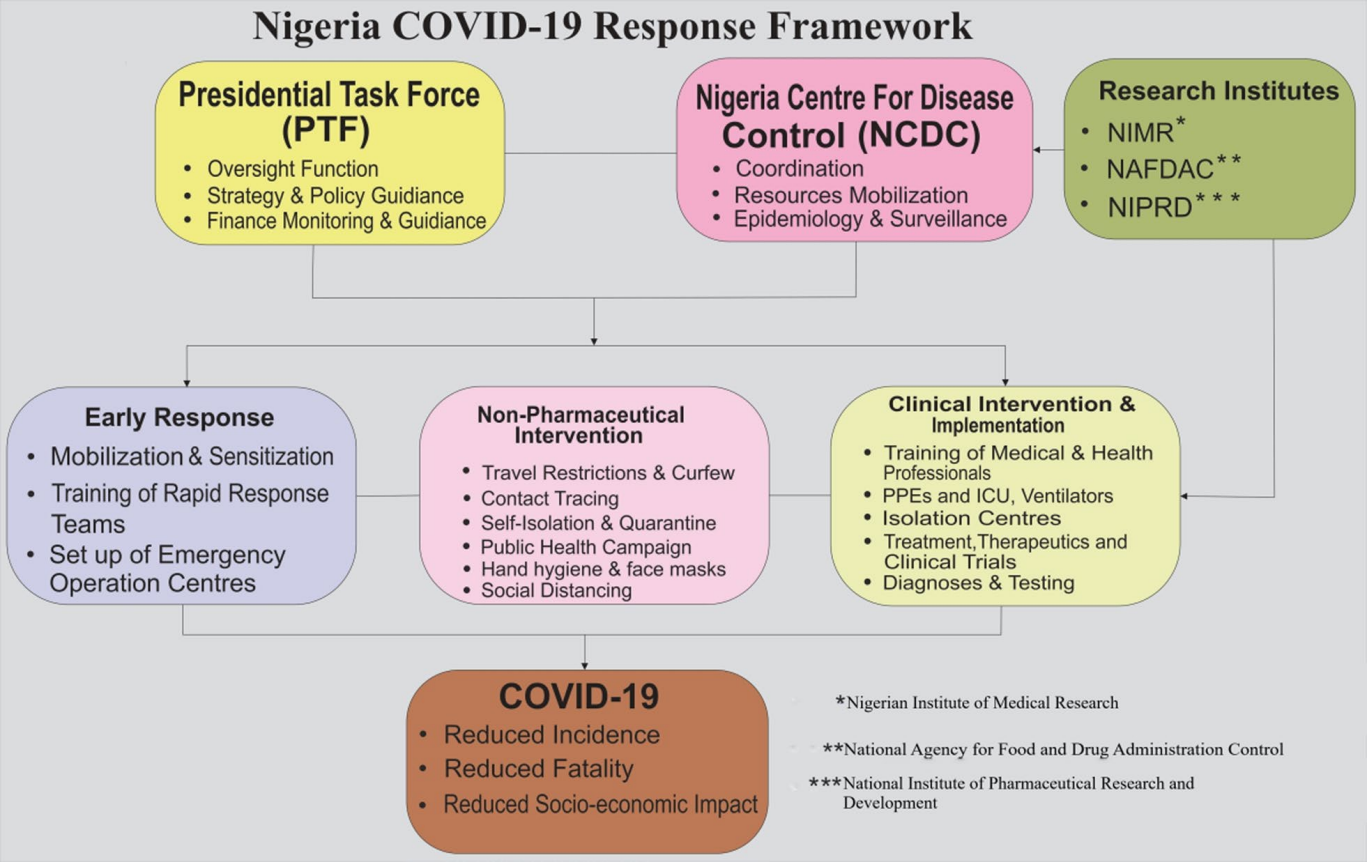
Nigeria COVID-19 response framework

Since the first index case of COVID-19 in Nigeria on February 27, 2020, Nigeria had implemented anti-COVID-19 mitigation and containment strategies. It also adopted a framework built on the existing infrastructural systems in the country as illustrated in Fig. 1. The framework is solely responsible for Nigeria’s response to the first wave of the COVID-19 epidemic. The National response framework was replicated at the state and local government levels.

In this paper, we critically evaluate Nigeria’s response to its first wave of COVID-19 from February 27 through November 30, 2020, against the backdrop of data-driven, evidence-based, and recommended public health measures.

## Main text

### Method

All 272 daily situation reports spanning February 27 through November 30, 2020, from the Nigeria Centre for Disease Control (NCDC), were analyzed and constitute the principal source of this review. A systematic literature search was also conducted to identify relevant articles that have evaluated COVID-19 response. Using PubMed and Google Scholar databases, terms related to “COVID-19 Nigeria”, “COVID-19 response Nigeria”, “COVID-19 NCDC” and “SARS-CoV-2 Nigeria” were combined and refined to include papers published from 2019; so as to limit the scope of this review to the most recent publications on COVID-19. Of the 864 articles retrieved, a total of 63 most relevant publications were used for this review. In estimating the instantaneous reproduction number (*R*), we used a framework developed by Cori et al. (2013) while adopting a mean serial interval of 3.96 days (95% confidence interval), and standard deviation of 4.75 days (95% confidence interval) (Du et al. 2020). The estimation was carried out for a period of 278 days, from February 27, through November 30, 2020. This was done for Nigeria, and for all its 36 states and the Federal Capital Territory (FCT).

## Results and discussion

### Epidemiology of COVID-19 in Nigeria

#### Morbidity, mortality, and recoveries

**Fig. 2:**
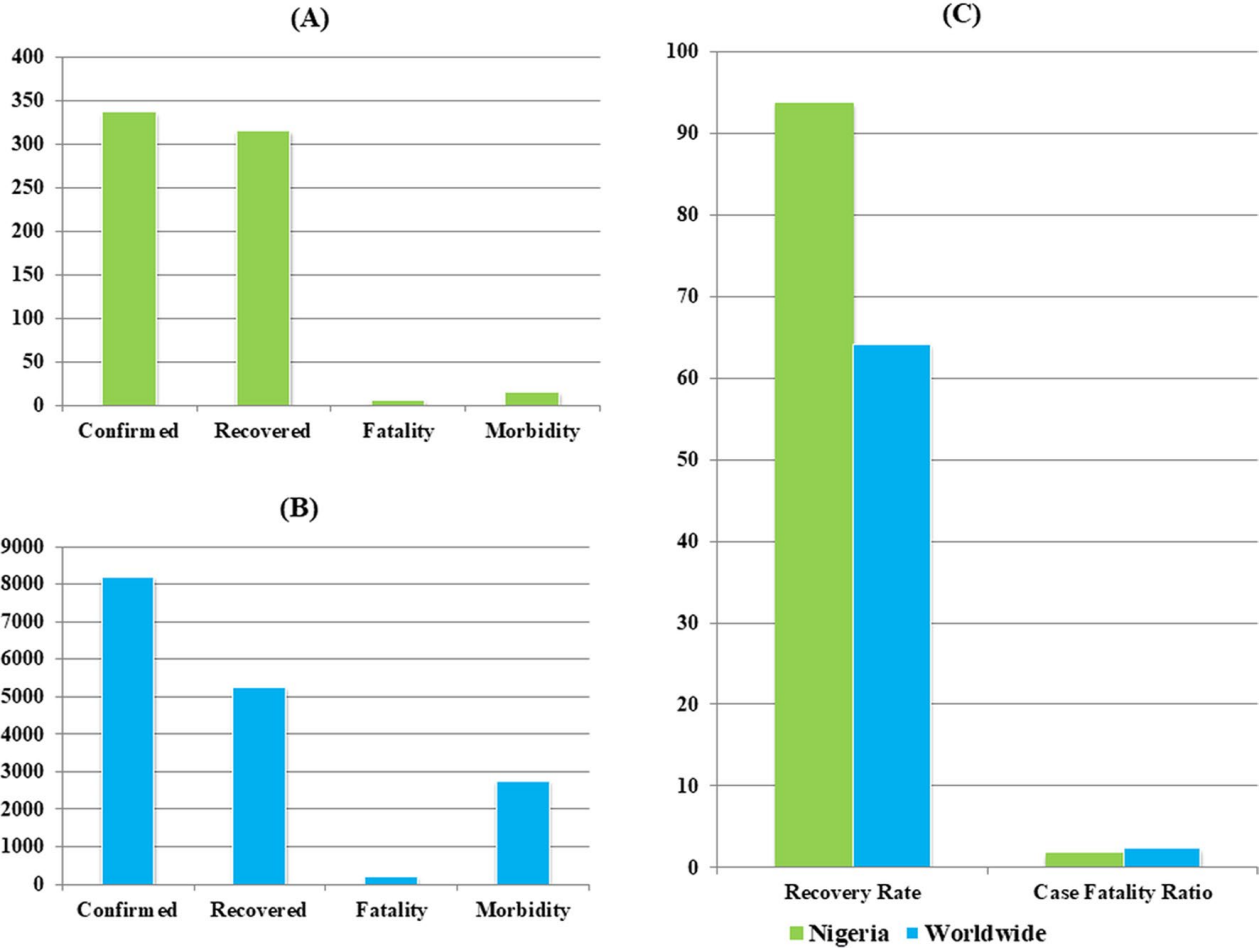
COVID-19 in Nigeria (NCDC 2020a) and worldwide (Worldometer 2020). (**a**), number of confirmed cases, recovered patients, death and morbidity, per million, in Nigeria,  (**b**), number of confirmed cases, recovered patients, death and morbidity, per million, worldwide, (**c**), rate of recoveries and case fatality ratio of COVID-19 world-wide and in Nigeria

The data on COVID-19 morbidities, mortality, and recoveries in Nigeria and worldwide, as of November 30, 2020, are presented in Fig. 2. The figure demonstrated that Nigeria has a milder COVID-19 epidemic compared to the global trend.

#### Virus reproduction numbers

The instantaneous reproduction number, (*R*) a measure of viral transmissibility, is the expected average number of secondary infectious cases produced by a primary infectious case over their infectious period (Cori et al. 2013). *R* calculated for Nigeria as of November 30, was 1.3 (95% CI). Further estimations were carried out for each of the 36 states of Nigeria and the FCT. There is no report from the NCDC on *R*, for Nigeria and various states, hence the reason *R* was calculated in this paper. *R* is an indispensable epidemic metric for monitoring the effectiveness of public health measures and since Nigeria has no published *R*, from NCDC, it is safe to assume that it was not used in decisions concerning non-pharmaceutical intervention measures. However, it cannot be discounted that NCDC/PTF may have used *R* to model the dynamics of the epidemic in publicly unavailable, internal position papers (Abubakar et al. 2021).

**Fig. 3:**
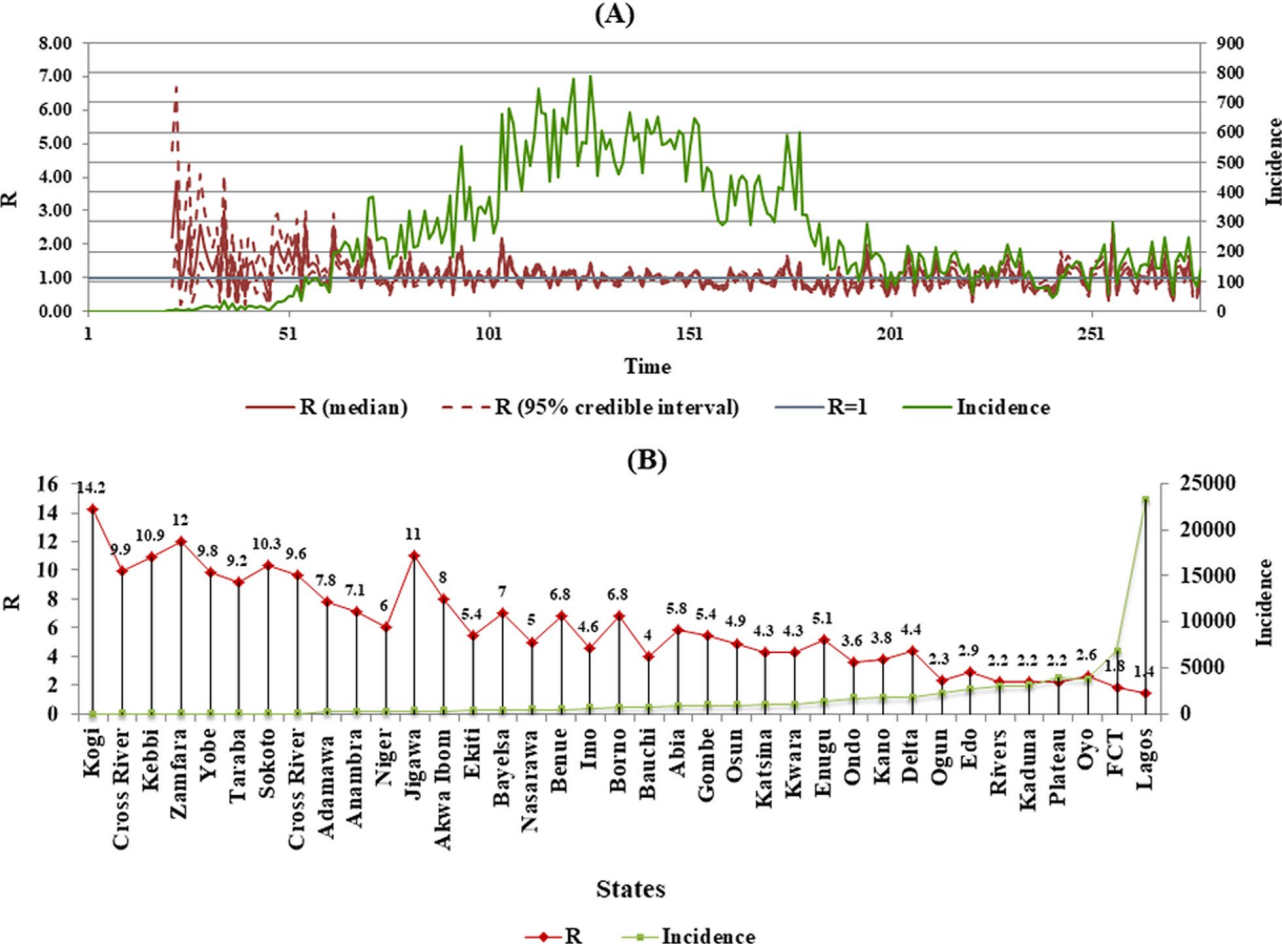
Instantaneous reproduction number (*R*) of SARS-CoV-2 in Nigeria: (**a**), *R* averaged over 278 days in Nigeria (Posterior median and 95% credible interval). (**b**), *R* for each of the 36 states and Federal Capital Territory (FCT) Abuja (95% credible interval)

It is however worthy of note that *R* does not capture the current status of an epidemic and may spike up and down when case numbers are low as evidenced in Fig. 3 where *R* seemed to spike when the daily cases were below 200 in the country (Adam 2020). This agrees with a report which estimated *R* for Lagos State to be 2.0 as of May 27, 2020, when the daily cases were less than 200 and the total incidence in the state, was 4012 (Okuonghae and Omame 2020). Moreso, *R* is an average for the total population and thus may mask local variation (Adam 2020), as is the case with Chile, Canada, and Brazil, which although presented lower *R* values during the same period, were still fighting to control the spread of the virus (Medina-Ortiz et al. 2020).

#### Risk factors and risk groups

The severe risks and complications associated with COVID-19 are relatively high for the population with underlying health conditions, while generally moderate for the general population. The other vulnerable populations are those with underlying health conditions such as diabetes, high blood pressure, cancer, liver disease, asthma, and tuberculosis (NCDC 2020b).

However, a study suggested that age is the largest risk factor for deaths due to COVID-19 (Rabi et al. 2020), although there is variation in the age group that is most affected worldwide. In Nigeria, age group 31–40 (29%) is the most affected, with fatality rate of 0.6%. In contrast, age 70 and older recorded the lowest incidence (2%), but with a death rate of 20%. Of the over 200 million inhabitants of Nigeria, only 3.1% make up the elderly population, of which 6.4 million are aged > 65 years. However, the NCDC guideline still regards persons aged 50 years and older, as vulnerable and at high risk of complications from COVID-19 (NCDC 2020b).

Nonetheless, another risk factor for COVID-19 mortality could be the male gender (Rabi et al. 2020), and so far, the male sex accounted for 63% (i.e. 39,676) of all infected cases, whereas the female sex accounted for 37% (i.e. 23,424) of total number of confirmed cases in Nigeria (NCDC 2020b).

## COVID-19 response: non-pharmaceutical interventions

### Travel restrictions and curfew

Travel restrictions into Nigeria were announced on March 18, 2020; 3 weeks after the first index case. This allowed enough time for the importation of the virus into the country, as returnees from abroad comprised the majority of those who tested positive for the virus (The Punch 2020a). Inter-state lockdown was first placed on three states with high incidence on March 29, and then on April 23, 2020, all the 36 states in the country and the FCT were placed on inter-state travel restriction, that is, 57 days after the index case was confirmed (The Punch 2020b). This response by the Nigeria’s government in imposing lockdown after 8 weeks was rather slow compared to countries like South Korea and Germany (Balmford et al. 2020), and this may have undermined conventional global COVID-19 prevention strategies and also indirectly aided community spread of the virus in the ensuing months in the country (Flaxman et al. 2020). Moreover, there is evidence that countries such as Germany and South Korea that imposed lockdown measures early on following the index cases or even before the index cases, were able to flatten the curve while countries such as the United States of America and the United Kingdom that did not introduce the lockdown measures early on witnessed virus exponential growth (Balmford et al. 2020). Although dusk-to-dawn curfew was enforced, the daily socio-economic activities of the citizens may have largely invalidated the impact the curfew was supposed to have on curtailing the spread of the virus by breaking the transmission chain (Ibrahim et al. 2020).

Nevertheless, the gradual ease of travel restriction/lockdown, first on May 4, 2020, than on June 6, 2020, was in disregard to World Health Organization guideline on ease of lockdown. As at the time when the lockdown measures began to be eased; the number of new infections was higher than number of recoveries, there was never 14-day fall in new infections and there was no evidence that showed that COVID-19 transmission is controlled, nor was the reproduction number less than 1 at any time in the country and in all the states (WHO 2020). Therefore, the ease of lockdown may have been majorly influenced by economic considerations and not out of regard for evidence-based epidemiological data. NCDC, the Nigeria’s public health authority argued that the ease of the lockdown measures at a time when COVID-19 cases was on the increase was a tradeoff aimed at balancing the public health concerns with the devastating economic consequences of the lockdown on Nigerians especially the most vulnerable including women, internally displaced persons, poor individuals, small and medium business enterprises among others (Dan-Nwafor et al. 2020).

### Social distancing measures

Social distancing is a strategy aimed at reducing physical contact between people, so as to reduce the risk and spread of COVID-19 in a community. This measure meant that, at least, two meters in physical distance must be maintained between two individuals. Moreso, physical greetings-hugs and handshakes were to be avoided (NCDC 2020c). In order to enforce this, the federal government of Nigeria prohibited large gatherings, issued compulsory stay-at-home directives to non-essential public servants, and also shut down schools, markets, and churches (NCDC 2020c). Not surprisingly, compliance with these directives was resisted by majority of the populace. In a country where the survival of over 85% of its population rely on their daily economic activities, even the meager palliatives given by the government could only reach about 2% of the population (Actionaid 2020), the bulk of the remaining 98% would inevitably starve to death if they were to abide by the social distancing measures. Faced with the grim choice of exposure to COVID-19 virus or hunger, most Nigerians choose to ignore social distancing measures in pursuit of their livelihood. This measure which failed in its enforcement may have sustained the transmission chain of the virus in states with high-density population such as Lagos, Oyo, Plateau, and FCT that recorded increased incidence of the virus.

### Source control measures

Measures taken in anticipatory bid to reduce likelihood of disease spread or prevent infected individuals from spreading disease are referred to as source control. These include but are not restricted to wearing face masks, hand hygiene, and respiratory hygiene (NCDC 2020d; MDH 2020). The NCDC advocated for the use of proper handwashing with soap and water, use of alcohol-based sanitizer, and respiratory hygiene when coughing or sneezing (NCDC 2020d). However, the use of face mask only received late attention after recommendation by Presidential Task Force (PTF) on April 27, 2020, a move that was then followed by the NCDC in May 4, 2020 (Abubakar et al. 2021), despite that there was scientific evidence from China, South-East Asia, and Europe as early as February/March 2020 that demonstrated the potency of face masks in reducing the spread of the virus (PAHO 2020). The NCDC decision on the use of face masks about 9 weeks after the first index case was rather improvident and may have further fueled the virus spread in the country.

### Self-isolation and quarantine measures

Self-isolation, defined by the NCDC to mean staying at home or in an identified accommodation, away from situations where one can mix with family members or the general public, for a period of 14 days, was also adopted as part of the measures to combat the virus. All returning travelers to Nigeria, anyone who had contact with a confirmed case, and COVID-19 patients who had just been discharged from the hospital were expected to self-isolate (NCDC 2020e). As is the case with South Africa, it is unclear how the quarantine process is being implemented as people self-isolate in homes (Moodley et al. 2020). Thus, the compliance level is difficult to estimate. This also means that the impact of this measure on Nigeria’s first wave COVID-19 response is inconclusive, but is however still subject to further investigation. However, it is important to note that prior to the ban on international travel, international passengers arriving at Nigerian international airports were allowed to self-quarantine for 14 days without testing and supervision by the Nigerian public health authority. Consequently, multiple undetected cases of COVID-19 may have been imported into Nigeria between January 2020 to March 18, 2020 (Dan-Nwafor et al. 2020). Expectedly NCDC with the introduction of travel ban migrated to obligatory supervised quarantine for all arriving passengers at Nigerian international airports and borders (Dan-Nwafor et al. 2020).

### Contact tracing

As of October 17, 2020, 34,901 were persons of interest in contact tracing, out of which 97.4% (33,994) have been traced. This is not so remarkable a feat because about 73% (44,483) of 56,557 confirmed cases were due to unknown source of exposure (NCDC 2020a). A likely explanation for this is that sustained community transmission of the virus has been ongoing prior to individuals testing positive for the virus. Moreover, the absence of a robust national health database in Nigeria means that contact tracing had to be done manually, which is rather slow and rely on the patient’s ability to recall. Furthermore, no attempt has been made in scaling up to a faster and more efficient digital tracing which seemed very effective when used in Taiwan (Wang et al. 2020b).

### Public health education campaign

The perception of the general populace is that they are at low risk for the disease, a misguided notion which was in part fueled by the poor handling of the early stage of the epidemic by the government agencies and health officials, as well as infodemics including the myths that Nigerians are immune to the virus and that COVID-19 is a fiction (Aiyewumi and Okeke 2020). Consequently, so as to sensitize the public on the virus transmission and infection dynamics, the NCDC released jingles, videos, and leaflets for public awareness on televisions, radio channels, and social media (NCDC 2020f). Although there is no independent evaluation of the effectiveness of these educational campaigns, it is likely that this move may have informed public acceptance of the other non-pharmaceutical intervention measures.

## COVID-19 response: clinical intervention and implementation

### Diagnosis and testing

The testing strategy in the current pandemic adopted by Nigeria has been the priority-based testing, where only individuals who show certain symptoms and, or have had contacts with any of the index cases, are tested for COVID-19. As of November 29, 2020, data from the NCDC showed that 776,768 tests had been carried out using the Reverse Transcriptase Polymerase Chain Reaction (RT-PCR) testing method. The absence of community testing capacity in Nigeria means that only a small fraction of the population was tested. In this same period, Nigeria (NCDC 2020a) conducted 3,865 tests per million of her population, which is very low in comparison with Ghana (GHS 2020) and Rwanda (RCS 2020) which had conducted at least 19,758 and 49,601 tests respectively, per million of their Population (World Bank 2020). This may be due to lack of rapid diagnostic kits, scarcity of reagents, and poor coordination among the 75 government diagnostic laboratories in the country (NCDC 2020g; Onyeaghala and Olajide 2020). The actual number of people infected is unknown, as seemingly healthy individuals were not tested unless they had travel history to high-index countries within a period range. Consequently, we suggest that the number of infected people, although asymptomatic, could have been more in Nigeria. Indeed, serological survey in October 2020 in four states in Nigeria detected antibodies to SARS-CoV-2 in 9–23% of individuals tested (Ihekweazu and Salako 2021). Thus COVID-19 cases, mortality, morbidity, and recovery may be grossly underestimated.

In addition, testing and diagnosis of COVID-19 were negatively impacted by political interference and inability to replicate the national response strategies at the state and local government levels. There seems to be little or no coordination between NCDC/PTF and the state governments, some state governors usurped the powers of members of the COVID-19 response framework and unilaterally introduced and implemented public health policies on COVID-19 without approval from NCDC or in disregard to them. This was the case in Kogi and Cross River states where the governors negatively impacted COVID-19 testing and control measures when they declared COVID-19 virus as hoax and prevented NCDC from implementing Covid-19 response measures (Premium Times 2020a).

### Treatments, therapeutics, and clinical trials

During this period, no effective antivirus treatment regimen for COVID-19 was available. Nevertheless, a recommendation by the Nigerian Institute of Medical Research (NIMR) advised the inclusion of any of Lopinavir, Ritonavir, or Ribavirin in the treatment of COVID-19 patients (NIMR 2020).

While preliminary reports have shown dexamethasone to be a promising drug in reducing mortality in COVID-19 patients, so far, there are clinical trials ongoing for at least, 10 other potential drugs of interest, including the Chloroquine (ACDC 2020). Meanwhile, Nigeria has not made any contribution to the development of a candidate vaccine, and the limited ongoing clinical trials are not the initiative of the Nigeria’s government but an off-shoot of WHO clinical trials.

### Isolation and treatment centers

At present, there is no official data or bulletin on the number and location of equipped structural facilities for the isolation and treatment of COVID-19 patients in the country. Nevertheless, a report by the Nigerian health minister gave an estimate of roughly 112 isolation centers with 5000 bed capacity, of which some of the 36 states in the country and the FCT have less than 300 beds for isolation and treatment of COVID-19 patients (Premium Times 2020b). A recent report stated that as of May 30 2020, Nigeria had 121 treatment centers with 6550 beds capacity (Dan-Nwafor et al. 2020). This translates to 32.7 beds per 1 million persons in Nigeria which is grossly inadequate for a country with a population of over 200 million human inhabitants. Thus, if infected, most Nigerians will not have access to isolation centers where they are less likely to infect other members of the society and receive proper treatment. An implication of this is that clinical assessment of morbidities and deaths due to the virus in Nigeria may have been largely underestimated.

### Availability of personal protective equipment, ventilators, intensive care units (ICUs)

While there has been no official data on the number of personal protective equipment, ventilators, and ICUs, that are available in Nigeria, it is estimated that there was 450 ventilators and 350 ICU beds at the start of the pandemic (Ogunbameru et al. 2020). For a country with a population of about 200 million persons, an extrapolation using the figure would mean that there were just about 2 ventilators per 1 million persons in Nigeria. Also, around the same period, the United Kingdom, with a population of about 67 million (World Bank 2020), had an estimated 8000 ventilators, which amounts to about 120 ventilators per 1 million persons (Pulse Nigeria 2020). The seemingly low number of deaths due to the SARS-CoV-2 virus may have obscured the dire need for more ICU beds in the country’s existing health facilities (Premium Times 2020c).

### Training of medical and health professionals

As part of the early response plan before the onset of the disease in Nigeria, the NCDC trained an undisclosed number of Rapid Response Teams (RRT) for early detection and effective response to public health emergencies, including Covid-19, across the 36 states of Nigeria and the FCT in December 2019. It is also worthy of note that the training was funded by the World Bank Regional Disease Surveillance System Enhancement project (REDISSE) (NCDC 2020h). Considering the low number of qualified health workers, the Nigeria’s government in May 2020, initiated the training of primary health care professionals in the country. In addition, NCDC had facilitated the training of over 17,000 health care workers on the principles of Infection Prevention and Control for COVID-19 via its e-learning platform as of July 2020. The impact of these training may be responsible for the low number (l540) of health care workers that as at then have contracted the disease in Nigeria (NCDC 2020i).

## Conclusions

Nigeria designed and implemented an integrated multi-sectoral response to COVID-19 epidemic. The containment and mitigation measures against COVID-19 seem at least in part to have dampened the virus transmission resulting in mild or moderate COVID-19 epidemic in Nigeria. Nigeria’s response to COVID-19 has been described as robust, aggressive, and a qualified success (Dan-Nwafor et al. 2020; Abubakar et al. 2021). However, the extent to which the response measures contributed to mild COVID-19 outcome is unknown as the viral, host, environmental, and public health factors that modulate the virus transmission dynamics, infection biology and pathophysiology are complex and largely unidentified. Except for the outlier South Africa, most countries in Sub-Saharan Africa had a mild or moderate COVID-19 epidemic (Adams et al. 2021) even though the response strategies to COVID-19 are not exactly the same. Nigeria response to the first wave of COVID-19 has some challenges and limitations namely: (1) absence of geo-mapping and electronic contact tracing capacity, (2) priority COVID-19 testing policy adopted by the government due to scarcity of sufficient testing resources and efficient laboratory networks, (3) lack of structural framework to address infodemics and the dearth of national research policy on pandemic preparedness, (4) premature easing of lockdown and social distancing measures at a time virus transmission is on the rise, (5) lack or unavailability of epidemiological data or models that inform decision making and response strategies in the public domain. (6) political interference that prevented NCDC from performing its statutory public health role, (7) poor implementation of the national response strategies especially at the local and community levels, and (8) the near absence of socio-economic support for families and businesses. These limitations have to be addressed and solutions proffered in order to optimize Nigerian response to subsequent phases or waves of the COVID-19 epidemic as well as for future pandemics.

## Data Availability

Data available on request from the authors.

## Abbreviations

COVID-19: Coronavirus Disease 2019
SARS-CoV-2: Severe Acute Respiratory Syndrome CoronaVirus-2
*R*: Instantaneous reproduction number
NCDC: Nigeria Centre for Disease Control
PTF: Presidential Task Force
FCT: Federal Capital Territory
WHO: World Health Organization
RT-PCR: Reverse Transcriptase Polymerase Chain Reaction
NIMR: Nigerian Institute of Medical Research
ICUs: Intensive Care Units
RRT: Rapid Response Teams
REDISSE: Regional Disease Surveillance System Enhancement
